# High Early Embryo Mortality and Low Hatching Success Observed in Aldabra Giant Tortoise Populations

**DOI:** 10.1002/ece3.73862

**Published:** 2026-06-28

**Authors:** Alessia Marialydia Lavigne, Richard Baxter, Eric Blais, Mark Brown, Robert Bullock, Christina Marques, Angelin Bernardis Sanders, Nirmal Jivan Shah, Chris Tagg, Elisabeth Wareing, James Wareing, Nicola Hemmings

**Affiliations:** ^1^ School of Biosciences, The University of Sheffield Sheffield UK; ^2^ Island Biodiversity Conservation Centre University of Seychelles & Indian Ocean Tortoise Alliance Victoria Seychelles; ^3^ Nature Seychelles Victoria Seychelles; ^4^ Cousine Island Company Limited Victoria Seychelles; ^5^ School of Life Sciences University of KwaZulu‐Natal Durban South Africa; ^6^ Save Our Seas Foundation D’Arros Research Centre (SOSF‐DRC), Save Our Seas Foundation Amirantes Seychelles; ^7^ North Island Company Limited Victoria Seychelles; ^8^ Island Conservation Society Victoria Seychelles

**Keywords:** Aldabra giant tortoise, climate change, conservation management, embryo mortality, population demographics, reproduction

## Abstract

Turtles and tortoises are highly threatened yet understudied, particularly regarding reproductive traits and early life stages. In long‐lived species, reliance on adult census data can mask reproductive failure for decades before population declines become apparent. This knowledge gap is evident in the Aldabra giant tortoises ( *Aldabrachelys gigantea* ), despite its widespread use in conservation translocations and ecosystem restoration. We present preliminary data on fertilisation success, early embryo survival and hatching success, across one natural and five translocated populations in the Seychelles. Of 317 eggs from 24 clutches, only 16% successfully hatched. Most failed eggs (97%) were undeveloped. Using recently developed microscopic methods to assess egg fertility, we provide the first population‐level comparisons of fertilisation and hatching outcomes for this species. Although sample sizes are limited, our results indicate low and variable hatching success, driven primarily by embryo mortality. Complete clutch failure was common (67%), and embryo mortality was prevalent even among clutches that had some degree of hatching success. Hatching rates were markedly reduced in translocated populations (0%–26%) compared to a natural Aldabra Atoll population (46%) and all fell below historical estimates from ~50 years ago (60%–80%). We also found a negative association between hatching success and a Human Modification Index, although interpretation of this result should be cautious considering the small sample size. These findings provide the first reproductive success data for translocated Aldabra giant tortoise populations and the first for the species in the last five decades. We argue that incorporating productivity metrics into monitoring frameworks is essential to improve vulnerability predictions and inform effective conservation management.

## Graphical abstract

This preliminary study provides the first population‐level assessment of fertilisation and hatching success in the Aldabra giant tortoise across natural and translocated populations in Seychelles, revealing low and highly variable hatching success—particularly in translocated populations. Most failed eggs were undeveloped, with early embryo mortality identified as the main cause of hatching failure. These findings suggest that relying solely on adult census data to monitor long‐lived species may mask declines in reproductive success, highlighting the urgent need to integrate monitoring of productivity and early‐life metrics (including fertilisation and hatching rates) into long‐term conservation programmes for long‐lived species.

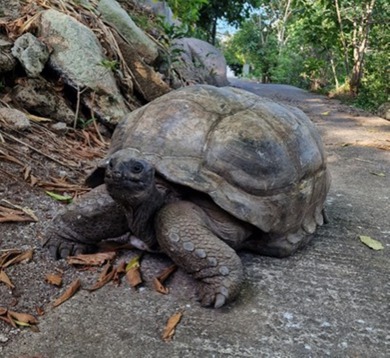

## 1 Introduction

More than half of all turtle and tortoise species or chelonians (Order: Testudines) are currently at risk of extinction (Rhodin et al. 2018). These long‐lived vertebrates are essential for ecological stability, serving as nutrient cyclers and ecosystem engineers (Lovich et al. 2018; Albertson et al. 2024). Despite being one of the most imperilled vertebrate groups, chelonians remain comparatively understudied, with key life‐history and ecological trait datasets being far less comprehensive than those available for other vertebrate groups (Wang et al. 2025). Data on chelonian early life stages and reproductive traits are scarce or entirely missing (Wang et al. 2025; Hunter et al. 2026). This knowledge gap is problematic, because understanding how these traits shape population dynamics is essential for predicting extinction risk and informing effective conservation strategies (Capdevila et al. 2022; Wang et al. 2025).

A major obstacle in chelonian conservation is the ‘perception of persistence’, a phenomenon where the presence of ageing adults gives a false impression of population health while recruitment has slowed or ceased (Lovich et al. 2018). While population growth rates are mathematically sensitive to adult survival, boosting the survival of eggs and juveniles may offer the greatest capacity for expanding the total number of individuals in a population (Knoerr et al. 2021; Souchay et al. 2013). However, because young turtles are often elusive (a period often called the ‘lost years’), baseline data for these stages are lacking for many species, complicating accurate threat assessments (Hunter et al. 2026).

**FIGURE 1:**
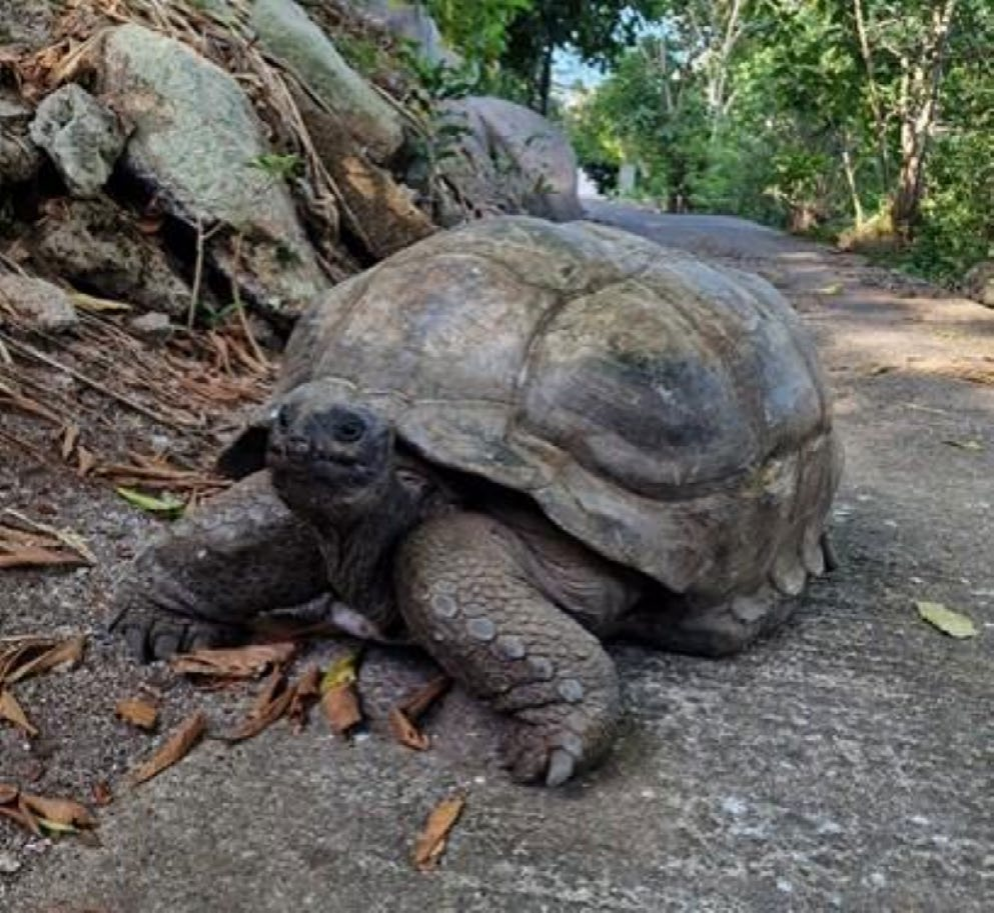
An Aldabra giant tortoise on Cousine Island, Seychelles.

The Aldabra giant tortoise ( *Aldabrachelys gigantea* ; Figure 1) epitomises this data gap; despite lifespans exceeding a century (Quesada et al. 2019), the last comprehensive studies of its recruitment occurred five decades ago (Swingland and Coe 1979; Gibson and Hamilton 1984). After facing near extinction in the late 19th century due to anthropogenic pressures (Stoddart et al. 1979), the only naturally occurring population now resides on Aldabra Atoll, comprising approximately 100,000 individuals (Bourn et al. 1999; Turnbull et al. 2015). Between 1850 and 1990, tortoises were actively translocated from Aldabra to various other Seychelles islands to safeguard the species against catastrophic events (Gerlach et al. 2013; Samour et al. 1987). In later years, further translocations were performed to also serve as ecological substitutes for extinct mega‐herbivores (Hansen et al. 2010; Gerlach et al. 2013; Stark and Galetti 2024). Although most free‐ranging tortoises in the Seychelles are believed to have Aldabran ancestry, the specific records of individual transfers, parental lineage and historical population distributions remain largely undocumented (Gerlach et al. 2013; Çilingir et al. 2022). The primary Aldabra atoll population appears stable (Turnbull et al. 2015), but numerous translocated populations across the Seychelles remain largely unassessed for reproductive viability. This lack of oversight is particularly concerning given that Aldabra giant tortoises are currently listed as ‘Vulnerable’ on the International Union for Conservation of Nature (IUCN) Red List (Tortoise and Freshwater Turtle Specialist Group 1996), and climate‐driven stressors, such as drought and habitat shifts, may disproportionately impact early life stages compared to adults (Haverkamp et al. 2017).

Determining the exact stage and drivers of recruitment failure in chelonian species is difficult, as early‐stage losses may occur in their cryptic post‐hatching ‘lost years’, or during the pre‐hatching (embryo death) or pre‐oviposition (fertilisation issues) stages (Lavigne et al. 2025; Hunter et al. 2026). Here, we addressed the latter by investigating the reproductive output of both a natural subpopulation of Aldabra and five introduced populations across the Seychelles. By employing novel microscopic techniques, we distinguish between failed fertilisation and early embryo mortality—two distinct drivers of recruitment loss (Lavigne et al. 2025). Our findings provide the first multi‐population comparison of tortoise nest productivity in the region, focusing on identifying potential recruitment failures that may be masked by the persistence of long‐lived adults and offering a vital update to the demographic data needed for effective management.

## 2 Materials and Methods

With the support of conservation partners, we obtained data on hatching success and egg fertility from as many clutches as possible in a 2‐year period across six Seychelles islands. In 2022/2023, samples were collected from Aldabra: Picard Island—a ~9.29 km^2^ coral island in the Aldabra Atoll which is a UNESCO World Heritage Site (Seychelles Islands Foundation; 9.3910° S, 46.2139° E); North Island—a small granitic island of ~2.01 km^2^ with an exclusive private resort (North Island Company Limited; 4.3950° S, 55.2453° E); and D'Arros Island—a privately owned small, flat, low lying coral sand cay of ~1.71 km^2^ (Save Our Seas Foundation: D'Arros Research Centre (SOSF‐DRC); 5.4180° S, 53.2962° E). In 2023/2024, samples were collected again from D'Arros Island, along with Cousin Island—a small ~0.3 km^2^ granitic island designated as a Special Reserve (Nature Seychelles; 4.3315° S, 55.6620° E); Cousine Island—a small exclusive granitic private island resort of ~0.3 km^2^ (Cousine Island Company Ltd.; 4.3507° S, 55.6475° E); and Desroches Island—a private coral island with an exclusive resort ~4.0 km^2^ coral sand cay (Island Conservation Society; 5.6952° S, 53.6583° E). We extracted Human Modification Indices (H‐values) for each study site (Table 1) from Theobald et al. (2025). H is a measure of the overall degree of human modification of an area, ranging from 0 (e.g., a very remote wilderness location), to 1 (a completely modified area).

**TABLE 1:** Clutch data from 24 different Aldabra giant tortoise clutches on a naturally occurring wild subpopulation of Aldabra Atoll (Picard Island) and five other Seychelles islands with translocated populations was used to calculate the number of clutches experiencing total clutch failure (i.e., 0% hatching success) as well as their average, range and median clutch size and hatching success.

	H value	Number of clutches	Mean clutch size (± standard error)	Number of total clutch failures	Mean % hatching success (all eggs)	% hatching success range (per clutch, min–max)	Median % hatching success (all eggs)
Aldabra Atoll (Picard)	0.001	7	10.3 (± 1.8)	2	46	0–91	44
Cousin	0.024	3	13.0 (± 3.5)	2	26	0–78	0
Cousine	0.025	3	16.3 (± 3.8)	3	0	0–0	0
D'Arros	0.210	4	17.0 (± 2.5)	2	11	0–36	3
Desroches	0.218	3	10.7 (± 2.3)	3	0	0–0	0
North Island	0.202	4	14.3 (± 3.5)	4	0	0–0	0
Total	—	24	13.6 (± 0.5)	16	19	0–91	0

*Note:* Data were collected from Aldabra and North Island during 2022/2023; Cousin, Desroches and Cousine from 2023/2024; and D'Arros samples were collected across both 2022/2023 and 2023/2024 nesting seasons. The overall degree of human modification was calculated for each location (H‐value) which ranges between values of 0 (e.g., for very remote wilderness area), to 1 (completely modified areas).

Partners identified as many tortoise nests (*n* = 24) as possible during the two breeding seasons, and at the end of incubation (~6–8 months), they excavated nests, collected eggs that showed no sign of embryonic development (i.e., undeveloped eggs) for fertility analysis, and recorded all egg fates. Due to significant challenges with identifying active Aldabra giant tortoise nests, our sampling approach for undeveloped eggs differed between the 2022/2023 and 2023/2024 breeding seasons. In 2022/2023, we aimed to sample 1–2 undeveloped eggs per clutch at random, from as many clutches as possible. However, since nest location proved difficult for our partners, in 2023/2024, we opted to collect as many undeveloped eggs as possible from any clutches found.

Where possible, partners counted the number of eggs in a nest during laying (without handling or otherwise manipulating eggs) to determine clutch size. Alternatively, clutch size was determined during post‐incubation excavations, and hatching success was calculated as the number of hatched eggs/total clutch size. We chose an incubation time of ~6–8 months to accommodate the variable time range of incubation between and within different populations, depending on environmental conditions. For example, on the Aldabra Atoll, reported incubation durations range from as little as 98 days (about 3 months) to 148 days (about 5 months; Swingland and Coe 1979), whilst some partners and zoos (e.g., Paignton and Barcelona Zoo) report incubation times up to ~8 months. Allowing an incubation period of ~6–8 months accommodates for this uncertainty and reduces the risk of mistaking any viable eggs as failed.

**FIGURE 2:**
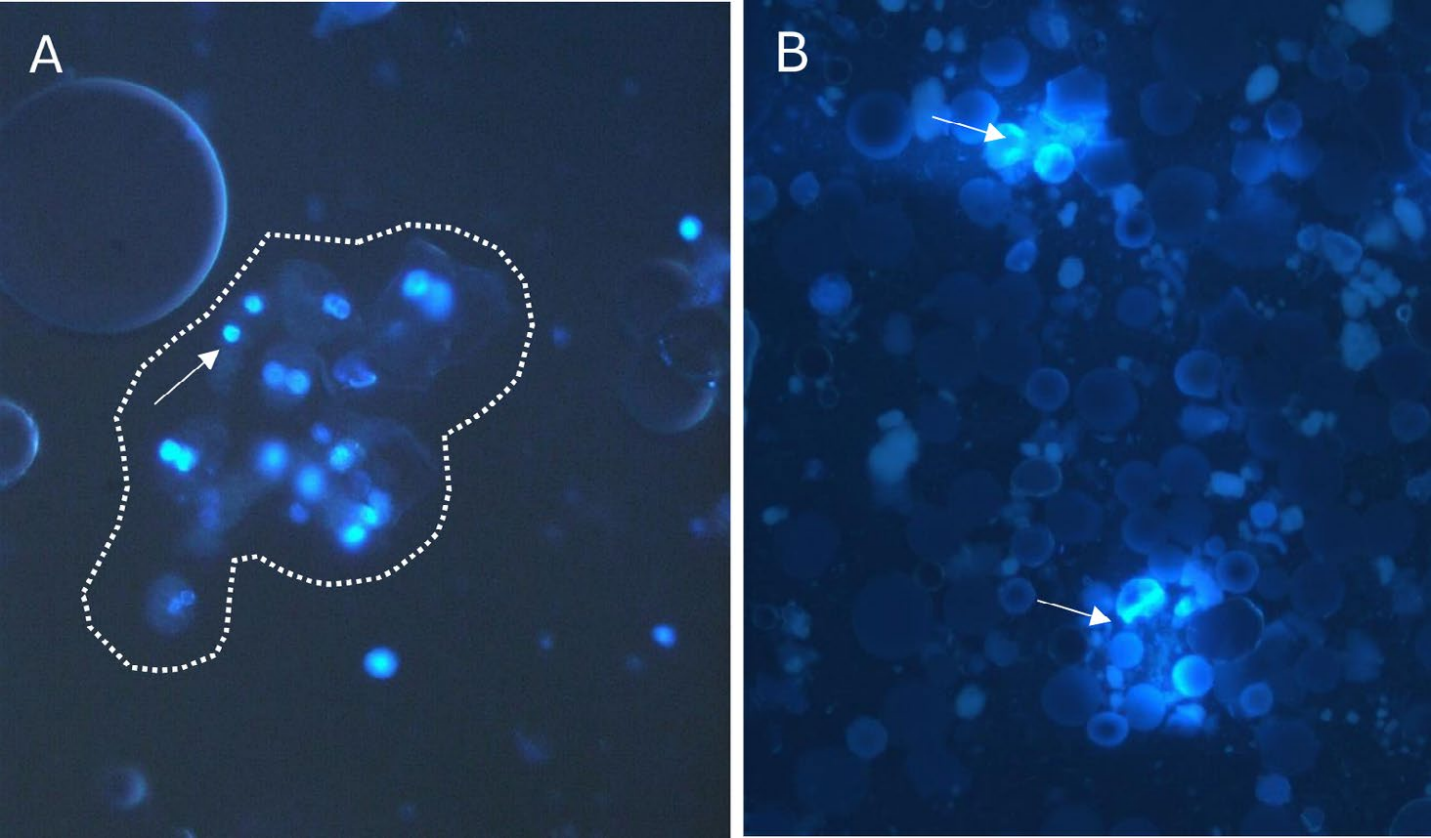
The fertility status of undeveloped Aldabra giant tortoise (Aldabrachelys gigantea) eggs is determined by the presence or absence of stained embryonic nuclei on the perivitelline membrane (PVM). (A) Microscopy image of a small cluster of embryonic cells at 200× magnification, indicated by the dotted outline. The embryonic nucleus of each cell (one is indicated by an arrow) is identifiable by its distinct, regular, round shape. Eggs with observed embryonic cells were recorded as ‘fertilised’. (B) Microscopy image of part of the PVM observed at 100× magnification without the presence of embryonic nuclei. The structures visible here are yolk globules which are larger, non‐fluorescent, cloudy and irregularly shaped. Auto‐fluorescent artefacts may occasionally be observed (indicated by arrows) but are easily distinguished from embryonic cell nuclei due to their structural characteristics. Whether an egg is considered unfertilised or inconclusive depends on how much intact PVM can be clearly analysed to ensure no embryonic cells are present in the sample.

All collected undeveloped eggs were stored in 5% formalin solution and transported to the University of Sheffield for laboratory analysis to determine their fertilisation status as described in Lavigne et al. (2025). Briefly, the undeveloped eggs were dissected and the perivitelline membrane (PVM) and/or germinal disc material were isolated and stained with the blue fluorescent, cell‐membrane permeant DNA stain Hoechst 33342 (Invitrogen) to enable the observation of embryonic nuclei and/or sperm cells. Samples were examined at 100–400× magnification, under a fluorescence microscope with UV illumination, a BP 340–380 excitation filter and LP 425 suppression filter (Figure 2). We considered eggs to be unfertilised if all or the majority of the PVM surrounding the yolk was retrieved (and the germinal disc if it had not deteriorated), but no embryonic nuclei and minimal/no sperm were found consistently across all PVM sections. In some cases, egg contents were very degraded due to being left in the nest for a long period of time. If the level of degradation meant we couldn't retrieve sufficient PVM to confidently determine fertility status (i.e., to clearly identify embryonic nuclei) and the germinal disc had deteriorated, we classified eggs as inconclusive.

### 2.1 Data Analysis

We tested for an association between the Human Modification Index and aggregated hatching rate per study site with a weighted binomial generalised linear model (GLM) fitted in R using the stats package (R Core Team 2026). The response variable was population‐level hatching outcome, specified as a two‐column matrix of the number of eggs hatched and not hatched, thereby modelling hatching success as a binomial process at the location level. We analysed hatching rates at the population rather than clutch level because the human modification index did not vary across clutches within a study site. Of the 317 eggs included in this analysis, 4 were unaccounted for and assumed to be not hatched.

### 2.2 Animal Ethics and Permits

This research was approved by the Seychelles Bureau of Standards to be carried out with Seychelles conservation organisations (Ref: A0157). Non‐viable Aldabra giant tortoise eggs were received opportunistically from Seychelles‐based collaborators and were authorised for export as per the agreement made with the Ministry of Agriculture, Climate Change and Environment, in accordance with Article 15 of the Convention of Biological Diversity. Additionally, permits were acquired under the Convention of International Trade in Endangered Species of Wild Fauna and Flora (CITES; Seychelles export permit #A1615, #A1622, #A1777; UK import permit #631966/03, #24GBIMPBRJYT2).

## 3 Results

In the 2022/2023 breeding season, partners collected 18 undeveloped eggs from 15 different naturally occurring clutches across Aldabra (Picard), North Island and D'Arros. In 2023/2024, partners collected 48 undeveloped eggs from 12 different clutches from D'Arros, Cousin, Desroches and Cousine (total of 66 undeveloped eggs from 27 clutches). Clutch data (i.e., information on clutch size and egg fates) were collected for 24 of these clutches.

We found hatching success varied considerably across Aldabra giant tortoise populations (Table 1) but appeared to be generally low, based on the clutches our partners found. On average, 67% (16/24) of monitored clutches suffered complete hatching failure, but success rates varied between islands, from 46% on a naturally wild subpopulation of Aldabra (Picard Island) to 0% on Cousine, Desroches and North Island (all of which are translocated populations). Out of 24 monitored clutches (total of 317 eggs), only 16% of eggs successfully hatched (52/317) and 82% (261/317) failed (4 eggs were unaccounted for). Most unhatched eggs were undeveloped (253/261 eggs), except for eight unhatched eggs across three clutches from Aldabra (Picard) that displayed visible signs of embryonic development. Therefore, undeveloped eggs accounted for 97% (253/261) of all failed Aldabra giant tortoise eggs. We also found a significant negative association between hatching success and human modification index (estimate = −15.78, SE ±2.99, *z* = −5.27, *p* < 0.001): across the six sites, those with a higher human modification index had higher rates of hatching failure.

**FIGURE 3:**
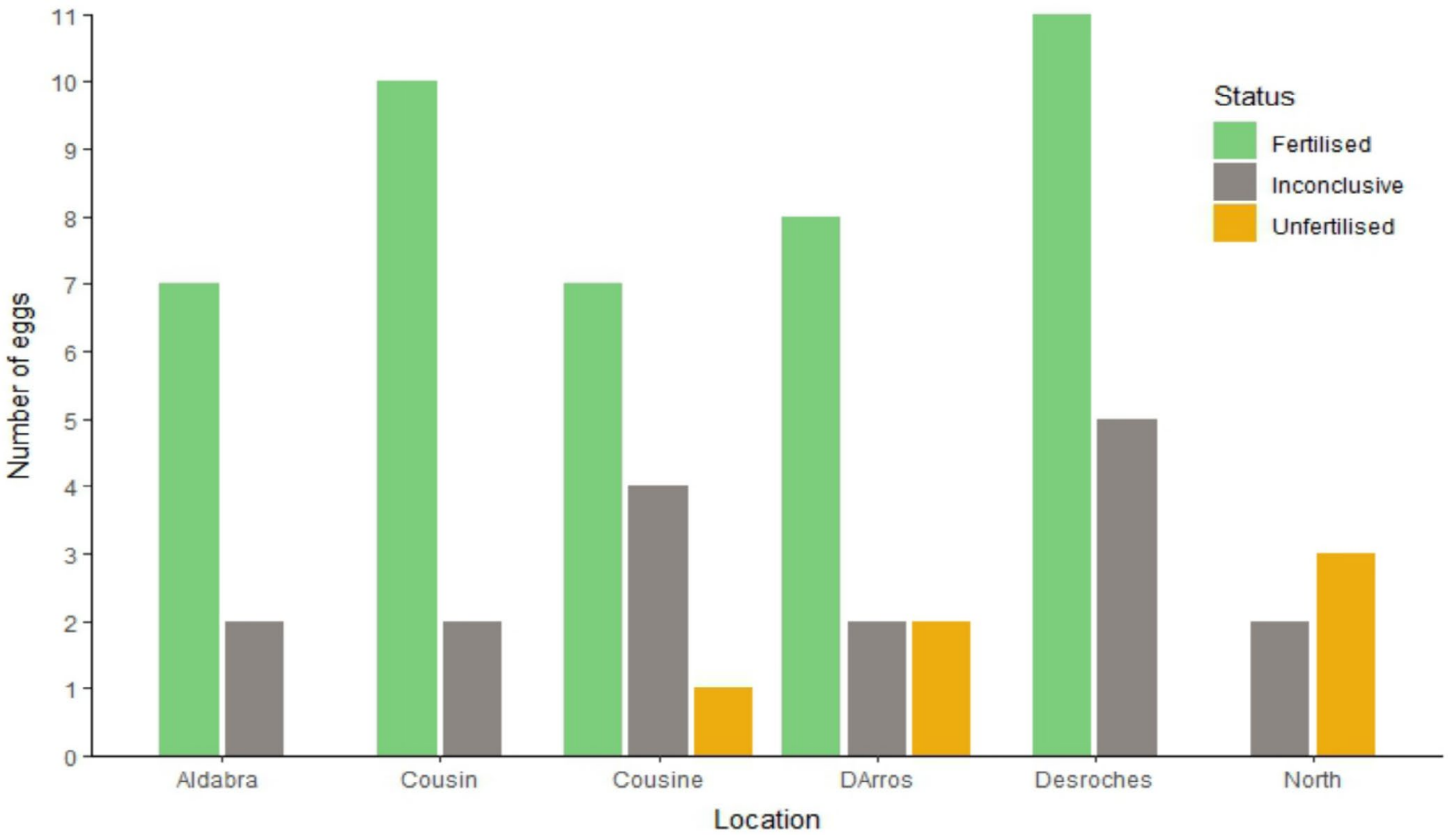
Fertility statuses of undeveloped eggs produced by Aldabra giant tortoises (Aldabrachelys gigantea, *n* = 66 eggs from 27 clutches) from six island locations in the Republic of Seychelles. Eggs were deemed inconclusive if there was insufficient material for further analysis. Samples were collected from Aldabra and North Island during 2022/2023; Cousin, Desroches and Cousine from 2023/2024; and D'Arros samples were collected across both 2022/2023 and 2023/2024 nesting seasons.

Across both breeding seasons, we determined the fertility status of 66 undeveloped eggs collected from 27 clutches across the six Seychelles islands (Figure 3). We found that fertilisation rates also varied across populations: all undeveloped egg samples that we were able to conclusively examine from Aldabra (natural population), Cousin and Desroches were fertilised, and most undeveloped eggs examined from Cousine and D'Arros were also fertilised, but we found no evidence of fertilisation in any conclusively examined undeveloped eggs from North Island. Therefore, in most populations, hatching failure in Aldabra giant tortoises is likely to be predominantly driven by embryo mortality, but fertilisation failure may pose a problem for some translocated populations (Figure 3 and Table 1).

## 4 Discussion

Our study provides the first population‐level discrimination between fertilisation failure and early embryo death in Aldabra giant tortoises. By applying novel microscopic techniques (Lavigne et al. 2025), we found that early embryo mortality is the first and primary barrier to hatching success across most Seychelles populations, rather than a lack of fertilised eggs. Due to challenges with locating nests, our data are limited and should be considered preliminary. However, they are nonetheless suggestive of very low hatching success across multiple populations, particularly those from translocated populations in this study. We argue that more detailed monitoring of hatching success, fertilisation rates and recruitment is needed to inform future management decisions.

While fertilisation failure was generally low, it appeared as a significant localised issue on North Island, where no examined eggs showed evidence of fertilisation in our sample (Figure 3). However, further investigation is warranted across multiple nesting seasons to fully understand these potential reproductive issues, since hatchlings, hatched eggs and dead embryos have occasionally been found on North Island in the past (although not reliably recorded/monitored over time; AS pers. obs.). The distinction between fertilisation failure and embryo mortality as causes of hatching failure is critical for management, as threats at the pre‐oviposition stage (such as sperm limitation or maternal health) require different interventions than pre‐hatching stressors like nest microclimate and pollution (Hunter et al. 2026). Future research into male sperm quality and the potential for multiple paternity—as seen in other chelonians (Lee and Hays 2024)—is needed to determine if fertilisation failures stem from mating system disruptions or physiological constraints. In addition, the impact of anthropogenic stressors on embryo survival and hatching success needs to be clarified: our data suggests that hatching success is lower in locations that have been more heavily modified by human activity, but this was based on limited data, and more extensive monitoring is needed to confirm this pattern and identify the specific anthropogenic drivers.

Our findings suggest a decline in tortoise productivity at the egg stage compared to historical benchmarks. However, determining a clear baseline of ‘normal’ hatching success is challenging due to the limited existing information. Current hatching success for the Aldabra atoll subpopulation on Picard (46%; Table 1) and translocated sites (0%–26%; Table 1) consistently falls well below the 60%–80% hatching rates reported 50 years ago on Aldabra (Malabar 60%, *n* = 213; Grande Terre 80%, *n* = 206; Swingland and Coe 1979). Our recorded mean clutch size on Picard (10.3 eggs, *n* = 7 clutches across one nesting season; Table 1) is also nearly 50% lower than the 19.2 eggs (*n* = 12 clutches across two nesting seasons) reported by Swingland and Coe (1979) in the same location. While these comparisons are based on limited temporal snapshots, the downward trend across multiple reproductive metrics—clutch size, fertility and hatching success—indicates a potential shift in the species' life‐history trajectory.

The observed high rates of embryo mortality may be a symptom of suboptimal incubation conditions driven by climate change. There is already evidence that environmental change can impact various components of productivity, including clutch size and number of clutches per female (Swingland and Coe 1979; Gibson and Hamilton 1984), and increased drought frequency and rainfall variability in the Seychelles have been posited to impact juvenile tortoises more severely than adults (Haverkamp et al. 2017). This highlights a dangerous ‘perception of persistence’ (Lovich et al. 2018; Hunter et al. 2026): because adult tortoises are exceptionally long‐lived and conspicuous, populations appear stable in censuses even when recruitment has functionally ceased. This phenomenon is not unique to *A. gigantea* ; similar potential cryptic declines and recruitment failures are currently being investigated in Galapagos giant tortoises (e.g., Western, *Chelonoidis porteri* , and Eastern, *C. donfaustoi*, Santa Cruz Galapagos tortoises; Blake et al. 2024), the only other extant giant tortoise taxa. By relying solely on adult counts, we risk masking population collapses until they are irreversible (Wheeler et al. 2003).

From a conservation perspective, to mitigate losses at the pre‐hatching stage we suggest integrating productivity metrics such as hatching and fertilisation success rates into standard monitoring frameworks. On islands like Cousin and Cousine, where natural hatching rates are currently near zero, exploring evidence‐based conservation interventions that may improve hatching success of fertilised eggs could provide a vital boost to recruitment. However, this approach must be paired with interventions such as habitat management to address the underlying causes of mortality (Hunter et al. 2026). Strategic research should prioritise identifying the specific microhabitat variables—such as soil moisture and temperature—that are driving the high rates of embryo death observed in this study.

In conclusion, the apparent stability of adult Aldabra giant tortoise populations may be a deceptive indicator of long‐term species health. While preliminary, our findings reveal a potential disconnect between adult presence and reproductive output, with current hatching rates falling well below the levels recorded five decades ago due to pervasive early embryo mortality. We encourage similar recent advances in understanding nesting ecology and recruitment, such as those demonstrated in the Galapagos giant tortoises (Blake et al. 2024), to be applied to Aldabra giant tortoise populations. However, addressing current knowledge gaps will require a greater investment from conservation programmes, particularly in the detection and monitoring of active nesting females and their clutches, which have currently not been systematically studied in recent times and most locations. Establishing long‐term monitoring frameworks that track active nests and productivity is therefore essential for generating robust baseline estimates of key demographic parameters, including hatching success and fertilisation rates. Only by identifying and mitigating these early‐life bottlenecks—whether through habitat‐focused climate mitigation or strategic interventions like headstarting—can we ensure that these persistent adult populations are successfully replaced by future generations.

## Author Contributions

**Alessia Marialydia Lavigne:** conceptualization (equal), data curation (lead), formal analysis (lead), funding acquisition (equal), investigation (lead), methodology (lead), project administration (lead), resources (supporting), validation (lead), visualization (lead), writing – original draft (lead), writing – review and editing (equal). **Richard Baxter:** writing – review and editing (supporting). **Eric Blais:** project administration (supporting). **Mark Brown:** project administration (supporting), writing – review and editing (supporting). **Robert Bullock:** investigation (supporting), project administration (supporting), writing – review and editing (supporting). **Christina Marques:** investigation (supporting), project administration (supporting). **Angelin Bernardis Sanders:** investigation (supporting). **Nirmal Jivan Shah:** project administration (supporting), resources (supporting). **Chris Tagg:** investigation (supporting). **Elisabeth Wareing:** investigation (supporting), project administration (supporting), writing – review and editing (supporting). **James Wareing:** investigation (supporting), project administration (supporting), writing – review and editing (supporting). **Nicola Hemmings:** conceptualization (equal), data curation (supporting), formal analysis (supporting), funding acquisition (equal), investigation (supporting), methodology (equal), project administration (supporting), resources (lead), supervision (lead), validation (supporting), visualization (supporting), writing – review and editing (equal).

## Funding

This work was supported by Seychelles Conservation and Climate Adaptation Trust (Blue Grants Fund small grant: 237) and Save Our Seas Foundation (SOSF small grant: 625), The Royal Society (Dorothy Hodgkin Research Fellowship DH160200 awarded to NH), and the Natural Environmental Research Council (NERC ACCE2 studentship NE/S00713X/1 awarded to AL).

## Conflicts of Interest

The authors declare no conflicts of interest.

## Supporting information


**Data S1:** Supporting information.

## Data Availability

The data that support the findings of this study are openly available in AGT_Dataset.csv (Supporting Information).
